# Advances in the Management of Children and Adolescents with Obesity

**DOI:** 10.3390/children12040452

**Published:** 2025-03-31

**Authors:** Manuel Reig García-Galbis, Rosa María Martínez-Espinosa

**Affiliations:** 1 Department of Analytical Chemistry, Nutrition and Food Science, Faculty of Sciences, University of Alicante, Carretera San Vicente del Raspeig s/n, 03690 Alicante, Spain; 2 Department of Biochemistry and Molecular Biology and Edaphology and Agricultural Chemistry, Faculty of Sciences, University of Alicante, Carretera San Vicente del Raspeig s/n, 03690 Alicante, Spain; rosa.martinez@ua.es

Obesity is a frequent, severe, complex, recurrent, and chronic disease characterized by an excessive accumulation of fat, which can have negative effects on health and decrease life expectancy. So far, obesity has been considered a public health problem [1].

This chronic disease was widely recognized as a major public health problem prior to the COVID-19 pandemic. The World Obesity Federation, in the fifth World Atlas of Obesity, predicts an increase in overweight and obesity from 2020 to 2035 that will affect more than 2.6 billion adults, children, and adolescents. Specifically, in boys and girls, an increase in prevalence of 10 to 20% and 8 to 18%, respectively, is expected [2].

Obesity occurs when a combination of genetic and epigenetic factors, risky behavioral patterns, and broader environmental and sociocultural influences affect body’s composition regulation systems [3,4]. Suggested treatments include behavioral interactions based on changes that the family should address in several areas: diet or dietary supplementation, physical activity, sedentary behavior, sleep quality, and drug treatment [5].

Although advances have been made in the treatment of obesity, the expected changes in overweight children and adolescents from low- and middle-income countries have not been observed as deserved. For this reason, this Special Issue was created to motivate experts to continue working on this topic, and to present new strategies such as lifestyle changes that lead to significant changes in body composition, biochemical markers, and other health parameters [6].

Among the works included in this Special Issue, there is an analysis of the global trends in the double burden of malnutrition (DBM) between the years 1990 and 2022, which concludes that there has been an increase in its prevalence in adults, adolescents, and children, due to the increase in cases of obesity [7].

As factors that contribute to a worsening of the health markers that determine obesity, in a study from Brazil, it has been observed that those living in the south of the country, attending public schools, and having the social context of higher education have a 1.94 [PR = 2.94 (95% CI: 1.20–7.23)] greater probability of developing DBM (Contribution 1). In addition, high consumption of beverages has been observed in children and adolescents when there is high consumption in parents, which contributes significantly to the development of obesity (Contribution 2). In a study conducted in Korea, it was observed that different types of social isolation in adolescents affect their levels of physical activity and, therefore, this social aspect has a direct effect on the development of obesity (Contribution 3).

As factors that contribute to an improvement in the health markers, it has been observed that family- and school-based interventions could be a therapeutic tool for these patients; however, more studies are needed to evaluate the effectiveness of new technologies based on tools such as the use of Smartphone Apps (Contributions 4–6).

Interventions based on the family and the school must be the fundamental pillars of future treatments for excess weight in children and adolescents and that is why this line of work must become a fundamental axis of research for obesity interventions in the coming years. In this area, works collected in this Special Issue recommend focusing special attention on adolescents between 13 and 17 years old, since this is the age range in which the greatest deficiency has been detected in research studies and, consequently, where the prevention, monitoring and treatment of obesity is currently least efficient. (Contributions 4 and 5) [8].
